# Pathologic response and survival after neoadjuvant chemotherapy with or without pertuzumab in patients with HER2-positive breast cancer: the Neopearl nationwide collaborative study

**DOI:** 10.3389/fonc.2023.1177681

**Published:** 2023-06-27

**Authors:** Agnese Fabbri, Fabrizio Nelli, Andrea Botticelli, Diana Giannarelli, Eleonora Marrucci, Cristina Fiore, Antonella Virtuoso, Simone Scagnoli, Simona Pisegna, Daniele Alesini, Valentina Sini, Armando Orlandi, Alessandra Fabi, Federico Piacentini, Luca Moscetti, Giuliana D’Auria, Teresa Gamucci, Marco Mazzotta, Laura Pizzuti, Patrizia Vici, Elisabetta Cretella, Paola Scavina, Annalisa La Cesa, Mara Persano, Francesco Atzori, Enzo Maria Ruggeri

**Affiliations:** ^1^ Department of Oncology and Hematology, Medical Oncology and Breast Unit, Central Hospital of Belcolle, Viterbo, Italy; ^2^ Department of Radiological, Oncological and Pathological Science, Sapienza University of Rome, Rome, Italy; ^3^ Biostatistics Unit, Scientific Directorate, Fondazione Policlinico Universitario A. Gemelli, IRCCS, Rome, Italy; ^4^ UOSD Centro Oncologico S. Spirito e Nuovo Regina Margherita (SS-NRM), Ospedale Santo Spirito in Sassia, Rome, Italy; ^5^ Department of Medical Oncology, Comprehensive Cancer Center, Fondazione Policlinico Universitario A. Gemelli IRCCS, Rome, Italy; ^6^ Precision Medicine Breast Unit, Scientific Directorate, Department of Women, Children and Public Health Sciences, Fondazione Policlinico Universitario Agostino Gemelli IRCCS, Rome, Italy; ^7^ Department of Medical Oncology, University Hospital of Modena, Modena, Italy; ^8^ Department of Medical Oncology, Medical Oncology Unit, Sandro Pertini Hospital, Rome, Italy; ^9^ UOSD Sperimentazioni di fase IV, IRCCS Regina Elena National Cancer Institute, Rome, Italy; ^10^ Department of Medical Oncology, Medical Oncology Unit, Ospedale Di Bolzano, Azienda Sanitaria Alto Adige, Bolzano, Italy; ^11^ Department of Medical Oncology, Medical Oncology Unit, San Giovanni Addolorata Hospital, Rome, Italy; ^12^ Department of Medical Oncology, Campus Bio-Medico University, Rome, Italy; ^13^ Department of Medical Oncology, University Hospital and University of Cagliari, Cagliari, Italy

**Keywords:** pertuzumab, trastuzumab, breast cancer, neoadjuvant chemotherapy, HER-2 positive, real-world data

## Abstract

**Purpose:**

Clinical trials have shown a significant increase in pathologic complete response (pCR) with the addition of pertuzumab to neoadjuvant chemotherapy for patients with early-stage HER-2 positive breast cancer. To date, limited studies have examined comparative outcomes of neoadjuvant pertuzumab in real-world setting. The Neopearl study aimed to assess comparative real-life efficacy and safety of neoadjuvant pertuzumab for these patients.

**Methods:**

We conducted a nationwide retrospective analysis involving 17 oncology facilities with a certified multidisciplinary breast cancer treatment committee. We identified patients with HER-2 positive stage II-III breast cancer treated with neoadjuvant chemotherapy based on trastuzumab and taxanes with or without pertuzumab. All patients underwent breast surgery and received a comprehensive cardiologic evaluation at baseline and after neoadjuvant treatment. Patients who received the combination of pertuzumab, trastuzumab, and chemotherapy constituted case cohort (PTCT), whereas those treated with trastuzumab and chemotherapy accounted for control cohort (TCT). The pCR rate and 5-year event free survival (EFS) were the primary outcomes. Secondary end-points were rates of conversion from planned modified radical mastectomy (MRM) to breast conservation surgery (BCS) and cardiotoxicities.

**Results:**

From March 2014 to April 2021, we included 271 patients, 134 (49%) and 137 (51%) in TCT and PTCT cohort, respectively. Positive axillary lymph nodes and stage III were more frequent in PTCT cohort. The pCR rate was significantly increased in patients who received pertuzumab (49% vs 62%; OR 1.74, 95%CI 1.04-2.89) and with HER-2 enriched subtypes (16% vs 85%; OR 2.94, 95%CI 1.60-5.41). After a median follow-up of 5 years, the 5-year EFS was significantly prolonged only in patients treated with pertuzumab (81% vs 93%; HR 2.22, 95%CI 1.03-4.79). The same analysis performed on propensity score matched population showed concordant results. On univariate analysis, only patients with positive lymph nodes were found to benefit from pertuzumab for both pCR and 5-year EFS. The rates of conversion from MRM to BCS and cardiologic toxicities did not differ between the cohorts.

**Conclusion:**

Our findings support previous data on improved outcomes with the addition of pertuzumab to trastuzumab-based neoadjuvant chemotherapy. This benefit seems to be more significant in patients with clinically positive lymph nodes.

## Introduction

Neoadjuvant chemotherapy (NACT) is a standard treatment option for early-stage breast cancer. The hoped-for outcome of NACT is a pathological complete response (pCR) on the surgical specimen, which is a viable surrogate for improved overall survival and a lower risk of relapse (1). NACT exerts the greatest potential in human epidermal growth factor receptor 2 (HER2) positive and triple-negative [estrogen receptor (ER) negative, progesterone receptor (PgR) negative, and HER2 negative] breast malignancies (2–4). Pertuzumab is a monoclonal antibody that targets the extracellular subdomain II of HER2 and thus inhibits ligand-dependent heterodimerization with other HER family members, including Epidermal Growth Factor Receptor (EGFR), HER3, and HER4. While the addition of pertuzumab to the combination of trastuzumab and chemotherapy in the advanced disease setting resulted in a pronounced increase in survival (5), the same therapeutic strategy produced only a marginal improvement of outcomes in the adjuvant context (6, 7). Dual HER2 blockade also improved the pCR rate significantly compared with trastuzumab alone when combined with NACT (8). Although subsequent clinical trials have confirmed the magnitude of the pCR rate (9–12), pertuzumab has been shown to be marginally beneficial in terms of survival outcome when combined with trastuzumab and NACT (13). This evidence has led international guidelines to recommend neoadjuvant pertuzumab as the standard of care, but preferably in patients with high-risk early breast cancer (14, 15). Since then, several retrospective series have evaluated the efficacy of neoadjuvant pertuzumab-based therapies in patients treated in real-world settings (16–19). Although these retrospective studies have confirmed the activity and toxicity profile of pertuzumab and narrowed the gap between the experimental setting and clinical practice, a comparison relying on real-life data with trastuzumab-based neoadjuvant treatment is still limited. We therefore conducted the Neopearl study, a real-world research that aimed to assess the efficacy and safety of trastuzumab and NACT with or without pertuzumab in a large cohort of early-stage HER2-positive breast cancer.

## Methods

### Study design and participants

The Neopearl is a multicenter, observational study involving 17 Italian oncology facilities. The study protocol follows the Strengthening the Reporting of Observational Studies in Epidemiology (STROBE) reporting guidelines and was formally registered (clinical trial identifier: EudraCT number 2022-001494-30). The referring Ethics Committee of the coordinating center (Central Hospital of Belcolle, Viterbo, Italy) and of each participating institution provided formal approval. The study was conducted in accordance with the Declaration of Helsinki, and all patients provided written informed consent. Anonymized demographic and clinical-pathological data, treatment details, and clinical outcomes were collected retrospectively through medical records. Eligible participants met the following inclusion criteria: age ≥18 years, histologically confirmed HER2-positive invasive breast cancer, localized extent of disease (stage II or III according to the American Joint Committee on Cancer’s Staging 8^th^ edition), at least two and no more than eight cycles of HER2-targeted NACT, adequate clinical reassessment before evaluation for breast surgery, and baseline and post-treatment estimation of left ventricular ejection fraction (LVEF). Evaluation of the extent of disease included breast magnetic resonance imaging before and after completion of NACT. Patients who received the combination of pertuzumab, trastuzumab, and chemotherapy constituted the experimental cohort (PTCT), whereas those treated with trastuzumab and chemotherapy accounted for the control cohort (TCT). After completion of NACT, a certified multidisciplinary committee from each participating institution discussed and confirmed the indication for surgery, which was performed according to international standards. Surgical resection was followed by adjuvant trastuzumab in the TCT cohort, trastuzumab and pertuzumab in the PTCT cohort, as well as radiotherapy and endocrine therapy as clinically indicated. As of March 2019, patients with residual invasive disease after NACT could also receive trastuzumab emtansine (T-DM1) (19).

### Procedures and assessments

All pathologic assessments were performed at each peripheral facility. Histological evaluation of pretreatment core biopsies established the diagnosis of invasive breast cancer. HER2-positive status was defined by either a score of 3+ by immunohistochemistry (IHC) or 2+ by IHC and positive fluorescent/chromogenic/silver *in situ* hybridization using the 2018 ASCO/CAP guidelines (20). ER and PgR status was determined by IHC according to local standards, with a cut-off ≥1% indicative of a positive result. The threshold value of a high expression for Ki67 IHC score was ≥20%. Pertuzumab (840 mg loading dose followed by 420 mg of each 21-day cycle) and trastuzumab (8 mg/kg loading dose followed by 6 mg/kg of each 21-day cycle or 4 mg/kg loading dose followed by 2 mg/kg weekly) were administered according to their labels. A taxane-containing chemotherapy regimen (docetaxel or paclitaxel) was chosen at the discretion of the prescribing physician. Dose and schedule modifications for toxicities were carried out per standard guidelines. Adverse events were defined and classified using the National Cancer Institute Common Terminology Criteria for Adverse Events (CTCAE version 5.0) (21). The primary end-points were the pCR rate, which was defined as no residual invasive or *in situ* tumor in the breast (ypT0/Tis) and axillary lymph node (ypN0) specimens after surgery (22), and event free survival (EFS), which referred to the time from the surgical resection to recurrence, metastasis, or death from any cause. Secondary end-points were the rate of conversion from planned modified radical mastectomy (MRM) at diagnosis to breast conservation surgery (BCS) and the incidence of cardiotoxicities. We also preplanned an exploratory analysis aimed at assessing the rate of pCR and EFS according to axillary lymph node status (clinically negative or positive). Patients who were not progressive or died were censored at the time of current analysis (cut-off date September 20, 2022). Cardiologic adverse events were defined as abnormalities of cardiac function, including decreased LVEF (reduction to ≤50% or more than 10% from baseline), congestive heart failure, and rhythm alterations.

### Statistical analysis

A mean with standard deviation was used to describe normally distributed variables, while a median with a 95% confidence interval (CI) or interquartile range (IQR) was reported for skewed variables. Comparative assessments were performed by applying Pearson’s *χ2* test for categorical data and Mann-Whitney *U* test for continuous variables. We planned a propensity score matching (PSM) to minimize potential selection bias between cohorts in case the distribution of prognostic factors showed an imbalance in favor of the experimental group. Propensity scores were calculated using a logistic regression model that included variables imbalanced between the cohorts. Matching was based on the nearest neighbor method with a ratio 1:1, without replacement and with a caliper of 0.1. A Fisher’s exact test was used to perform a univariate analysis of the correlation between clinical variables and pCR. A multivariate logistic regression model was performed to estimate the odds ratio (OR) of pCR with a 95% CI as a function of significant variables in the univariate analysis. A Mantel-Cox log-rank test was used to compare the survival outcomes of different patient subgroups according to significant variables. Survival curves were visualized through the Kaplan-Meier method. A multivariate Cox regression model was applied to estimate the hazard ratio (HR) with a 95% CI of variables included in the univariate survival analysis. All tests performed were two-sided, and a *p* value <0.05 was considered significant. SPSS (IBM SPSS Statistics for Windows, version 23.0, Armonk, NY) and Prism (GraphPad, version 9) software were used for statistical evaluations and figure rendering, respectively. R software version 4.1.2 and library MatchIt were used for PSM.

## Results

### Patient characteristics

From March 2014 to April 2021, the study included 271 consecutive eligible patients, 134 (49%) and 137 (51%) of whom had received trastuzumab and chemotherapy (TCT) or their combination with pertuzumab (PTCT), respectively. The distribution of clinical and pathological features was mostly homogeneous across the treatment cohorts with the exception of clinically positive axillary lymph nodes and stage III disease, which were significantly more frequent in PTCT subgroup. Since both variables are thought to be adverse prognostic factors (4), we performed an initial analysis including the general population without performing PSM because an imbalance in favor of the experimental cohort was clearly unlikely. An additional evaluation based on PSM was conducted to confirm the first-level results (**Supplementary Figure 1**). **Table 1** details baseline patient characteristics. All patients had an LVEF greater than 55%, indicative of adequate cardiac function before the initiation of NACT. Comparative evaluation also showed that significantly more patients received a weekly schedule, paclitaxel-based, and anthracycline-containing NACT regimen in TCT cohort. As expected, the duration of NACT was shorter in PTCT cohort. Conversely, the duration of adjuvant chemotherapy was longer in the same subgroup (**Table 2**).

**Table 1:** Baseline patient characteristics.

	General population			PSM population		
	TCT cohort (N=134)	PTCT cohort (N=137)	P value	TCT cohort (N=83)	PTCT cohort (N=83)	P value
Age - mean, years (SD) - <40 years	51.9 (12.3) 23 (17.2%)	51.8 (11.2) 19 (13.9%)	0.921 0.454	53.8 (13.2) 14 (16.9%)	50.3 (10.7) 13 (15.7%)	0.056 0.833
Menopausal status - premenopausal - postmenopausal	72 (53.7%) 62 (46.3%)	62 (45.3%) 75 (54.7%)	0.163	44 (53.0%) 39 (47.0%)	41 (49.4%) 42 (50.6%)	0.641
BMI - mean (SD) - >25	24.37 (4.91) 72 (53.7%)	26.15 (5.28) 69 (50.4%)	0.231 0.579	22.96 (4.62) 42 (50.6)	27.70 (5.50) 36 (53.4%)	0.649 0.351
ECOG PS - 0 - 1	113 (84.3%) 21 (15.7%)	125 (91.2%) 12 (8.8%)	0.082	66 (79.5%) 17 (20.5%)	75 (90.4%) 8 (9.6%)	0.051
Histology - ductal - lobular - other	128 (95.5%) 6 (4.5%) -	132 (96.3%) 3 (2.2%) 2 (1.5%)	0.22	77 (92.8%) 6 (7.2%) -	81 (97.6%) 2 (2.4%) -	0.147
T stage - T1 - T2 - T3 - T4	15 (11.2%) 78 (58.2%) 30 (22.4%) 11 (8.2%)	12 (8.8%) 83 (60.6%) 25 (18.2%) 17 (12.4%)	0.533	11 (13.3%) 42 (50.6%) 20 (24.1%) 10 (12.0%)	7 (8.4%) 47 (56.6%) 17 (20.5%) 12 (14.5%)	0.661
Nodal status - N0 - N1 - N2 - N3	86 (64.2%) 37 (27.6%) 11 (8.2%) -	35 (25.5%) 72 (52.5%) 27 (19.7%) 3 (2.2%)	<0.001	35 (42.2%) 37 (44.6%) 11 (13.3%) -	35 (42.2%) 37 (44.6%) 11 (13.3% -	1
Stage group - II - III	97 (72.4%) 37 (27.6%)	78 (56.9%) 59 (43.1%)	0.008	47 (56.6%) 36 (43.4%)	47 (56.6%) 36 (43.4%)	1
Grading - 2 - 3	35 (26.1%) 99 (73.9%)	36 (26.3%) 101 (73.7%)	0.976	23 (27.7%) 60 (72.3%)	24 (28.9%) 59 (71.1%)	0.863
ER status - negative - positive	47 (35.1%) 87 (64.9%)	61 (44.5%) 76 (55.5%)	0.112	31 (37.3%) 52 (62.7%)	33 (39.8%) 50 (60.2%)	0.750
PgR status - negative - positive	79 (58.9%) 55 (41.1%)	87 (63.5%) 50 (36.5%)	0.442	48 (57.8%) 35 (42.2%)	48 (57.8%) 35 (42.2%)	1
Ki 67 expression - <20% - ≥20%	6 (4.5%) 128 (95.5%)	12 (8.8%) 125 (91.2%)	0.157	3 (3.6%) 80 (96.4%)	7 (8.4%) 76 (91.6%)	0.192
HER2 status - 3+ - 2+	103 (76.9%) 31 (23.1%)	103 (75.2%) 34 (24.8%)	0.536	66 (79.5%) 17 (20.5%)	55 (66.3%) 28 (33.7%)	0.055
Planned surgery - BCS - MRM	62 (46.3%) 72 (53.7%)	69 (50.4%) 68 (49.6%)	0.500	31 (37.3%) 52 (62.7%)	40 (48.2%) 43 (51.8%)	0.158

PSM, propensity score matching; TCT, trastuzumab-chemotherapy; PTCT, pertuzumab-trastuzumab-chemotherapy; SD, standard deviation; BMI, body mass index; ECOG PS, Eastern Cooperative Oncology Group Performance Status; ER, estrogen receptor; PgR, progesterone receptor; HER-2, human epidermal growth factor receptor 2; BCS, breast conservation surgery; MRM, modified radical mastectomy.

**Table 2:** Neoadjuvant and adjuvant therapy features in the general population.

	TCT cohort (N=134)	PTCT cohort (N=137)	P value
Duration of NACT, weeks (median, 95% CI)	21.3 (20.0-21.5)	18.5 (17.7-20.5)	<0.001
Antracycline-containing NACT	127 (94.8%)	86 (62.8%)	<0.001
Carboplatin-containing NACT	10 (7.5%)	4 (2.9%)	0.091
Taxane-based NACT - paclitaxel - docetaxel	126 (94.0%) 8 (6.0%)	84 (61.3%) 53 (38.7%)	<0.001
Taxane schedule of NACT - weekly - every three weeks	98 (73.1%) 36 (26.9%)	36 (26.3%) 101 (73.7%)	<0.001
Duration of adjuvant therapy, weeks (median, 95% CI)	30.7 (30.5-31.1)	33.5 (31.5-34.4)	<0.001
Any adjuvant therapy	128 (95.5%)	123 (89.8%)	0.070
Antracycline-containing adjuvant therapy	7 (5.2%)	15 (10.9%)	0.084
Adjuvant trastuzumab emtansine	3 (2.2%)	9 (6.6%)	0.083

TCT, trastuzumab-chemotherapy; PTCT, pertuzumab-trastuzumab-chemotherapy; NACT, neoadjuvant chemotherapy; CI, confidence interval.

### Pathologic response

All patients included in the Neopearl study underwent surgical resection and were evaluable for pathologic assessment. The median time to breast surgery from the start of NACT was significantly shorter in patients treated with pertuzumab [TCT cohort 25.5 weeks (95% CI 25.1-25.8) vs. PTCT cohort 23.0 weeks (95% CI 22.0-24.3); p<0.001]. One hundred and fifty-one patients met the criteria for pCR in the general population [55.7% (95% CI 49.8-62.0)]. Univariate analysis revealed that negative ER and PgR status, HER2 3+ score at IHC, and treatment with pertuzumab were significantly associated with an increased pCR rate. Only the last two variables retained their positive predictive significance in the multivariate testing. The PSM population-based evaluation confirmed these findings (**Table 3**). In the exploratory analysis, the pCR rate did not differ within the subset of patients with clinically negative lymph nodes [TCT cohort 55.8% (95% CI 45.3-66.3) vs. PTCT cohort 51.4% (95% CI 37.1-68.6), p=0.691]. Significantly more patients achieved a pCR after treatment with pertuzumab in the subgroup with clinically positive lymph nodes [TCT cohort 37.5% (95% CI 25.0-52.1) vs. PTCT cohort 65.7% (95% CI 56.9-75.5), p=0.001]. The conversion rate from planned MRM to BCS did not differ between the treatment subsets (TCT cohort 8.2% vs. PTCT cohort 8.0%, p=0.924).

**Table 3:** Analysis of correlation between clinical-pathological variables and pCR.

Variable	General population					PSM population				
	Univariate analysis			Multivariate analysis		Univariate analysis			Multivariate analysis	
	no pCR N=120 (100%)	pCR N=151 (100%)	P value	OR (95% CI)	P value	no pCR N=83 100%)	pCR N=83 (100%)	P value	OR (95% CI)	P value
Age - <40 years - ≥40 years	13 (10.8%) 107 (89.2%)	29 (19.2%) 122 (88.8%)	0.065	–	–	9 (10.8%) 74 (89.2%)	18 (21.7%) 65 (78.3%)	0.058	–	–
Menopausal status - premenopausal - postmenopausal	54 (45.0%) 66 (55.0%)	80 (53.0%) 71 (47.0%)	0.222	–	–	39 (47.0%) 44 (53.0%)	46 (55.4%) 37 (44.6%)	0.277	–	–
BMI - ≤25.00 - >25.00	52 (43.3%) 68 (56.7%)	78 (51.6%) 73 (48.3%)	0.181	–	–	38 (45.8%) 45 (54.2%)	50 (60.2%) 33 (39.8%)	0.062	–	–
ECOG PS - 0 - 1	106 (88.3%) 14 (11.7%)	132 (87.4%) 19 (12.6%)	0.854	–	–	71 (85.5%) 12 (14.5%)	70 (84.3%) 13 (15.7%)	0.828	–	–
Nodal status - negative - positive	55 (45.8%) 65 (54.2%)	66 (43.7%) 85 (56.3%)	0.806	–	–	33 (39.8%) 50 (60.2%)	37 (44.6%) 46 (55.4%)	0.530	–	–
Stage group - II - III	72 (60.0%) 48 (40.0%)	103 (68.2%) 48 (31.8%)	0.263	–	–	45 (54.2%) 38 (45.8%)	49 (59.0%) 34 (41.0%)	0.263	–	–
ER status - negative - positive	37 (30.8%) 83 (69.2%)	71 (47.0%) 80 (53.0%)	0.009	0.78 (0.41-1.50)	0.470	23 (27.7%) 60 (72.3%)	41 (49.4%) 42 (50.6%)	0.004	0.40 (0.20-0.80)	0.009
PgR status - negative - positive	62 (51.7%) 58 (48.3%)	104 (68.9%) 47 (31.1%)	0.006	0.61 (0.32-1.16)	0.132	42 (50.6%) 41 (49.4%)	54 (65.1%) 29 (34.9%)	0.059	–	
Ki 67 expression - <20% - ≥20%	8 (6.7%) 112 (93.3%)	10 (6.6%) 141 (93.4%)	1	–	–	6 (7.2%) 77 (92.8%)	4 (4.8%) 79 (95.2%)	0.514	–	–
HER2 status - 2+ - 3+	41 (34.2%) 79 (65.8%)	24 (15.9%) 127 (85.1%)	<0.001	2.94 (1.60-5.41)	<0.001	32 (38.6%) 51 (61.4%)	13 (15.7%) 70 (84.3%)	<0.001	3.58 (1.60-8.01)	0.002
Treatment cohort - TCT - PTCT	68 (56.7%) 52 (43.3%)	66 (49.2%) 85 (62.0%)	0.038	1.74 (1.04-2.89)	0.032	46 (55.4%) 37 (44.6%)	37 (44.6%) 46 (55.4%)	0.162	1.98 (1.01-3.92)	0.049

pCR, pathologic complete response; PSM, propensity score matching; OR, odds ratio; CI, confidence interval; BMI, body mass index; ECOG PS, Eastern Cooperative Oncology Group Performance Status; ER, estrogen receptor; PgR, progesterone receptor; HER-2, human epidermal growth factor receptor 2; TCT, trastuzumab-chemotherapy; PTCT, pertuzumab-trastuzumab-chemotherapy.

### Survival

**Figure 1:**
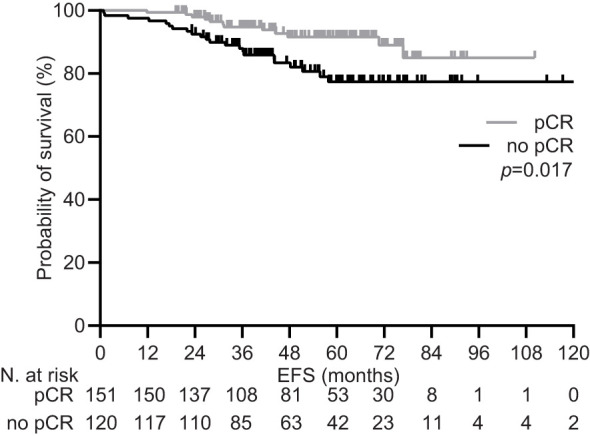
Event free survival by pathologic response in the general population pRC, pathologic complete response.

**Figure 2:**
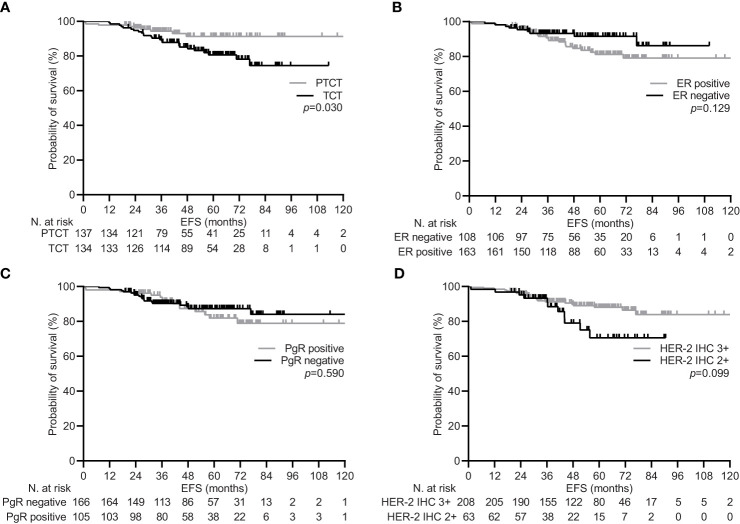
Event free survival depending on significant clinical variables in the general population. **(A)** type of neoadjuvant treatment: pertuzumab-trastuzumab-chemotherapy (PTCT) vs trastuzumab-chemotherapy (TCT); **(B)** estrogen receptor (ER) status: positive vs negative; **(C)** progesterone receptor (PgR) status: positive vs negative; **(D)** human epidermal growth factor receptor 2 (HER-2) scoring at immunohistochemistry (IHC): 3+ vs 2+.

**Figure 3:**
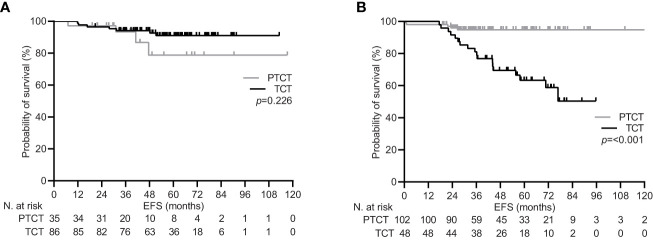
Event free survival by lymph node status in the general population. **(A)** clinically lymph node negative disease (cN0); **(B)** clinically lymph node positive disease (cN+). PTCT, pertuzumab-trastuzumab-chemotherapy; TCT, trastuzumab-chemotherapy.

During a median follow-up of 59.5 months (95% CI 53.2-65.9), 34 (12.5%) patients experienced disease recurrence, including one case of local relapse. Achieving pCR was confirmed to be associated with a significant increase in the 5-year EFS rate within the general population [92.1% (95% CI 87.4-96.0) vs. 81.7 (74.2-88.3), p=0.017; **Figure 1**]. Univariate subgroup analysis showed a significant survival benefit only for patients treated with pertuzumab. This was confirmed in the multivariate testing, with no significant interaction found across other subgroups (**Table 4** and **Figure 2**). In the subset of patients with negative lymph node disease, treatment with pertuzumab did not confer a significant advantage in the 5-year EFS rate [91.9% (95% CI 84.9-97.7) vs. 88.6 (77.1-97.1), p=0.226; **Figure 3A**]. However, the survival benefit of pertuzumab was more pronounced in patients with lymph node positive disease [62.5% (95% CI 47.9-77.1) vs. 95.1 (91.2-99.0), p<0.001; **Figure 3B**]. The same analyses performed on the PSM-based population showed concordant results (**Table 4**; **Supplementary Figures 1**, **2**).

**Table 4:** Analysis of correlation between clinical-pathological variables and EFS.

Variable	General population				PSM population			
	Univariate analysis		Multivariate analysis		Univariate analysis		Multivariate analysis	
	5-year EFS rate (95% CI)	P value	HR (95% CI)	P value	5-year EFS rate (95% CI)	P value	HR (95% CI)	P value
ER status - negative - positive	91.7% (86.1-96.3) 84.7% (79.1-89.6)	0.129	0.66 (0.30-1.45)	0.309	88.5% (80.5-96.5) 74.8% (64.4-85.2)	0.378	1.00 1.54 (0.55-4.29)	0.411
PgR status - negative - positive	88.6% (83.7-92.8) 85.7% (79.0-91.4)	0.590	0.92 (0.46-1.83)	0.825	82.6% (74.0-91.2) 76.0% (63.5-88.5)	0.832	1.00 0.77 (0.31-1.94)	0.583
HER2 status - 2+ - 3+	82.5% (73.0-90.5) 88.9% (84.6-93.3)	0.073	1.83 (0.89-3.79)	0.099	66.0% (46.0-86.0) 82.8% (75.0-90.6)	0.277	1.00 0.60 (0.26-1.36)	0.221
Treatment cohort - TCT - PTCT	81.3% (74.6-88.1) 93.4% (89.1-97.1)	0.030	2.22 (1.03-4.79)	0.041	74.1% (64.1-84.1) 87.4% (76.6-98.2)	0.038	1.00 0.37 (0.15-0.93)	0.035

EFS, event-free-survival; HR, hazard ratio; PSM, propensity score matching; CI, confidence interval; ER, estrogen receptor; PgR, progesterone receptor; HER-2, human epidermal growth factor receptor 2; TCT, trastuzumab-chemotherapy; PTCT, pertuzumab-trastuzumab-chemotherapy.

### Safety

All participants underwent at least one cardiologic evaluation after NACT and were evaluable for cardiotoxicity. The incidence of cardiologic adverse events of all grades and serious adverse reactions did not differ significantly between the cohorts. The main manifestations of cardiotoxicity incleded ST-segment abnormalities, sinus tachycardia, and a transient decrease in LVEF. Hematologic toxicities were more frequent in the TCT cohort, but only neutropenia showed a significantly increased incidence. Similarly, patients in the TCT cohort more commonly experienced other non-hematologic toxicities, which did not differ significantly. **Table 5** details adverse events with an incidence ≥5%.

**Table 5:** Most common adverse events in the general population.

	TCT cohort (N=134)	PTCT cohort (N=137)	P value
Cardiologic events, any grade	6 (4.4%)	14 (10.2%)	0.070
Cardiologic events, ≥ grade 3	2 (1.5%)	5 (3.6%)	0.262
LVEF decline	10 (7.5%)	14 (10.2%)	0.424
Anemia, any grade	53 (39.6%)	39 (28.5%)	0.054
Anemia ≥ grade 3	6 (4.5%)	1 (0.7%)	0.051
Neutropenia, any grade	63 (47.0%)	30 (21.9%)	<0.001
Neutropenia ≥ grade 3	21 (15.7%)	7 (5.1%)	0.004
Febrile neutropenia	4 (3.0%)	2 (1.5%)	0.393
Thrombocytopenia, any grade	15 (11.2%)	9 (6.6%)	0.180
Thrombocytopenia ≥ grade 3	2 (1.5%)	1 (0.7%)	0.548
Diarrhea, any grade	47 (35.1%)	39 (28.5%)	0.242
Diarrhea, ≥ grade 3	2 (1.5%)	4 (2.9%)	0.424
Other, any grade	43 (32.1%)	32 (23.3%)	0.108
Other, ≥ grade 3	6 (4.5%)	9 (6.5%)	0.540

TCT, trastuzumab-chemotherapy; PTCT, pertuzumab-trastuzumab-chemotherapy; LVEF, left ventricular ejection fraction.

Other adverse events included peripheral sensory neuropathy, nausea, mucositis, asthenia, and decreased appetite.

## Discussion

We reported the results of a comparative analysis in terms of pCR, EFS, and safety on the addition of pertuzumab to trastuzumab and taxane-based regimens as neoadjuvant chemotherapy for HER-2-positive early breast cancer. Accordingly, patients treated with pertuzumab in the real-world setting achieved a significantly higher pCR rate of 62%, which compares favorably with the same figure reported in the pivotal NeoSphere trial (8). The pCR rate in our series is widely consistent with that described by several phase II and III clinical trials (9–12) and other large observational studies of real-world data in non-Asian populations (16–18, 23). A more appropriate evaluation of this result could be performed with two studies with a similar design of nonrandomized comparison (24, 25). The percentage of patients with a pCR after surgery and the relative increase compared with control treatment were again consistent with our data. In contrast to what was reported in both studies, our multivariate analysis showed a benefit in pCR rate independent of hormone receptor status but larger in the subgroup of patients with HER-2 IHC 3+ malignancies. The latter result finds confirmation in translational studies suggesting that non HER-2-enriched subtypes are less sensitive to HER-2 dual blockade (26). We also described a significant benefit for this end-point in patients with positive lymph nodes, based on a preplanned univariate analysis. Although this finding is not evident in similar studies, its significance seems to be consistent with the more pronounced benefit of adjuvant pertuzumab in the same subgroup of patients (6, 7).

Our survival analysis showed that adding pertuzumab provides a significant advantage in terms of EFS at 5 years, with an absolute increase of more than 12%. Although this difference appears particularly pronounced, the percentage of patients free of disease-related events is comparable to the figures reported for neoadjuvant trastuzumab- and pertuzumab-based NACT at the same time point (12–19). It is worth noting that treatment with pertuzumab was the strongest predictive factor in the multivariable testing, demonstrating a beneficial effect independent of other potential predictors. However, our univariate analysis revealed that pertuzumab was associated with a 5-year EFS benefit only in patients who had positive lymph nodes at the time of diagnosis. This finding is consistent with previous neoadjuvant data and outcomes of adjuvant pertuzumab treatment (6, 7, 13).

The safety analysis in our series was mainly aimed at evaluating cardiotoxicities. Although no significant differences were found between the two treatment groups, the incidence of cardiologic adverse events of any grade and severe grade increased twofold in patients given pertuzumab. This finding is even more relevant considering that a significantly lower proportion of patients in the same cohort received an anthracycline-containing regimen. Previously published real-world comparative studies have not evaluated cardiologic safety, making even an indirect comparison inconsistent. However, a comparable proportion of patients in both cohorts had a 10% reduction in LVEF from baseline or its decline below 50%, and none experienced symptomatic left ventricular systolic dysfunction. These data are concordant with the results of clinical trials (10, 27–30) and meta-analytic assessment (31).

Therefore, comparative evaluation with available evidence confirms a high efficacy and favorable toxicity profile of neoadjuvant pertuzumab in patients treated outside the experimental setting. The methodological approach adopted in the Neopearl study has inherent strengths and weaknesses. Despite clinical trials provide the most compelling level of evidence, they only involve less than 5% of cancer patients, raising concerns about the validity of experimental outcomes in the population treated in clinical practice (32). Real-world studies have become an essential part of cancer research, as they provide different stakeholders with data that can bridge the distance between clinical trials and routine practice (33). The main strength of our study is the reliability of collected data. Our results relied on nationwide collaboration of institutions with a certified disease-oriented multidisciplinary committee (InterBreast Network). The validity of the medical records in our retrospective series is ensured by their close matching with the registry of the government agency for monitoring high-cost drug prescriptions (including trastuzumab and pertuzumab) (34). In addition, the use of pertuzumab was based on the availability of the drug at each individual facility rather than on individual patient characteristics. This prescriptive attitude, together with the variety of facilities involved, meant that most prognostic factors were evenly distributed across the cohorts under study. However, lymph node-positive status and stage III disease were more frequently represented in patients given pertuzumab. The choice to conduct a first-level analysis on the general population without a PSM including these variables may be controversial, but it seems clear that such statistical variation in the sample would have had an unfavorable prognostic impact on patients treated with trastuzumab alone. Moreover, it would have compromised the ability of our case series to reflect prescriptive attitudes in clinical practice. Second-level analysis based on a homogeneously distributed sample after PSM can further confirm the reliability of our findings.

The Neopearl study acknowledges further limitations. Although our results relied on rigorous multivariable testing, the possibility of residual bias remained inherent in its retrospective observational design. The small number of events and the resulting low statistical power did not allow us to perform an analysis of overall survival. The latter consideration emphasizes the need for a longer follow-up period in ours, as in most real-world studies. We also found significant differences in chemotherapy regimens and their duration between patients treated with and without pertuzumab. Because anthracycline-containing schedules were largely limited to patients receiving only trastuzumab, additional confounding by treatment indication cannot be ruled out.

## Conclusions

The Neopeal is the first multicenter observational study conducted in Italy on the efficacy and safety of neoadjuvant pertuzumab in HER-2 positive early-stage breast cancer. Our results strengthen the evidence from experimental and real-world studies that the addition of pertuzumab can result in a significant improvement in the pCR rate and EFS with a safe toxicity profile. However, approximately half of the patients in our series achieved a pCR without receiving pertuzumab. This finding further confirmed that a sizeable proportion of them could obtain an excellent outcome from neoadjuvant treatment with trastuzumab and taxane-based chemotherapy alone (35). Further research on predictive biomarkers is warranted to identify subgroups of patients who might benefit most from the addition of pertuzumab to standard NACT (36). In this regard, our exploratory analysis indicate that patients with lymph node positivity and HER-2-enriched subtypes are the best candidates for neoadjuvant dual HER-2 blockade.

## Data availability statement

The raw data supporting the conclusions of this article will be made available by the authors, without undue reservation.

## Ethics statement

The studies involving human participants were reviewed and approved by Comitato Etico Lazio 1 (protocol number for approval 957/CE Lazio1). The patients/participants provided their written informed consent to participate in this study.

## Author contributions

AgF, FN, AB and ER conceptualized the study. DG performed the statistical analysis. EM, CF, AV, SS, SP, DA, VS, AO, AlFA, FP, LM, GD’A, TG, MM, LP, PV, EC, PS, AC, MP, and FA collected clinical and setup the database. AgF and ER provided resources for the study. AgF, FN, and ER supervised the study. AgF, FN and AB wrote initial draft and edited final version. All authors contributed to the interpretation of the data. All authors discussed the results and critically revised the manuscript. All authors read and approved the final manuscript.
