# Supplementary material for: Pathologic response and survival after neoadjuvant chemotherapy with or without pertuzumab in patients with HER2-positive breast cancer: the Neopearl nationwide collaborative study
Source: Front Oncol. 2023 Jun 27;13:1177681. doi: 10.3389/fonc.2023.1177681 (PMC10335743; doi:10.3389/fonc.2023.1177681)
Supplement: Supplementary file 1 [file DataSheet_1.pdf]

SUPPLEMENTARY MATERIAL

Pathologic response and survival after neoadjuvant chemotherapy with or without pertuzumab in patients with HER2-positive breast cancer: The Neopearl nationwide collaborative study.

Agnese Fabbri[1†], Fabrizio Nelli[1†], Andrea Botticelli[2*], Diana Giannarelli[3], Eleonora Marrucci[1], Cristina Fiore[1], Antonella Virtuoso[1], Simone Scagnoli[2], Simona Pisegna[2], Daniele Alesini[4], Valentina Sini[4], Armando Orlandi[5], Alessandra Fabi[6], Federico Piacentini[7], Luca Moscetti[7], Giuliana D'Auria[8], Teresa Gamucci[8], Marco Mazzotta[8], Laura Pizzuti[9], Patrizia Vici[9], Elisabetta Cretella[10], Paola Scavina[11], Annalisa La Cesa[12], Mara Persano[13], Francesco Atzori[13], and Enzo Maria Ruggeri[1], on behalf of the Interbreast Network

[†] These authors contributed equally to this work and share first authorship

[1] Department of Oncology and Hematology, Medical Oncology and Breast Unit, Central Hospital of Belcolle, Viterbo, Italy

[2] Department of Radiological, Oncological and Pathological Science, Sapienza University of Rome, Rome, Italy

[3] Biostatistics Unit, Scientific Directorate, Fondazione Policlinico Universitario A. Gemelli, IRCCS, Rome, Italy

[4] UOSD Centro Oncologico S. Spirito e Nuovo Regina Margherita (SS-NRM), Ospedale Santo Spirito in Sassia, Rome, Italy

[5] Department of Medical Oncology, Comprehensive Cancer Center, Fondazione Policlinico Universitario A. Gemelli IRCCS, Rome, Italy

[6] Precision Medicine Breast Unit, Scientific Directorate, Department of Women, Children and Public Health Sciences, Fondazione Policlinico Universitario Agostino Gemelli IRCCS, Rome, Italy

[7] Department of Medical Oncology, University Hospital of Modena, Modena, Italy

[8] Department of Medical Oncology, Sandro Pertini Hospital, Rome, Italy

[9] Department of Medical Oncology 2, Istituti di Ricovero e Cura a Carattere Scientifico (IRCCS) Regina Elena National Cancer Institute, Rome, Italy

[10] Department of Medical Oncology, Ospedale Di Bolzano, AS Alto Adige, Bolzano, Italy

[11] Department of Medical Oncology, San Giovanni Addolorata Hospital, Rome, Italy

[12] Department of Medical Oncology, Campus Bio-Medico University, Rome, Italy.

[13] Department of Medical Oncology, University Hospital and University of Cagliari, Cagliari, Italy.

* **Correspondence:**
Andrea Botticelli, MD; Department of Radiological, Oncological and Pathological Science, Sapienza University of Rome, Rome, Italy; andrea.botticelli@uniroma1.it

Supplementary Figure 1
Supplementary Figure 2
Supplementary Figure 3

Supplementary Figure 1. Covariate balance before and after propensity score matching

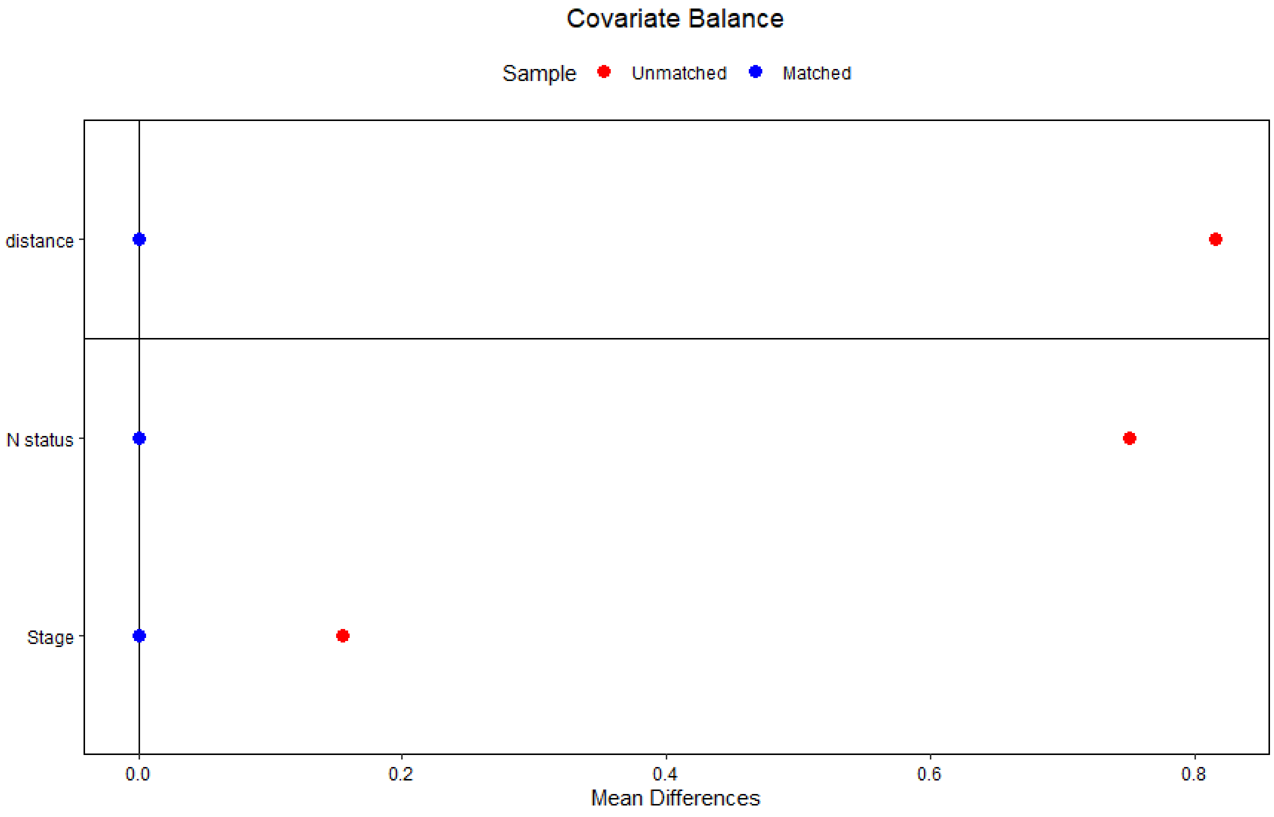

Supplementary Figure 2. Event free survival depending on significant clinical variables after PSM

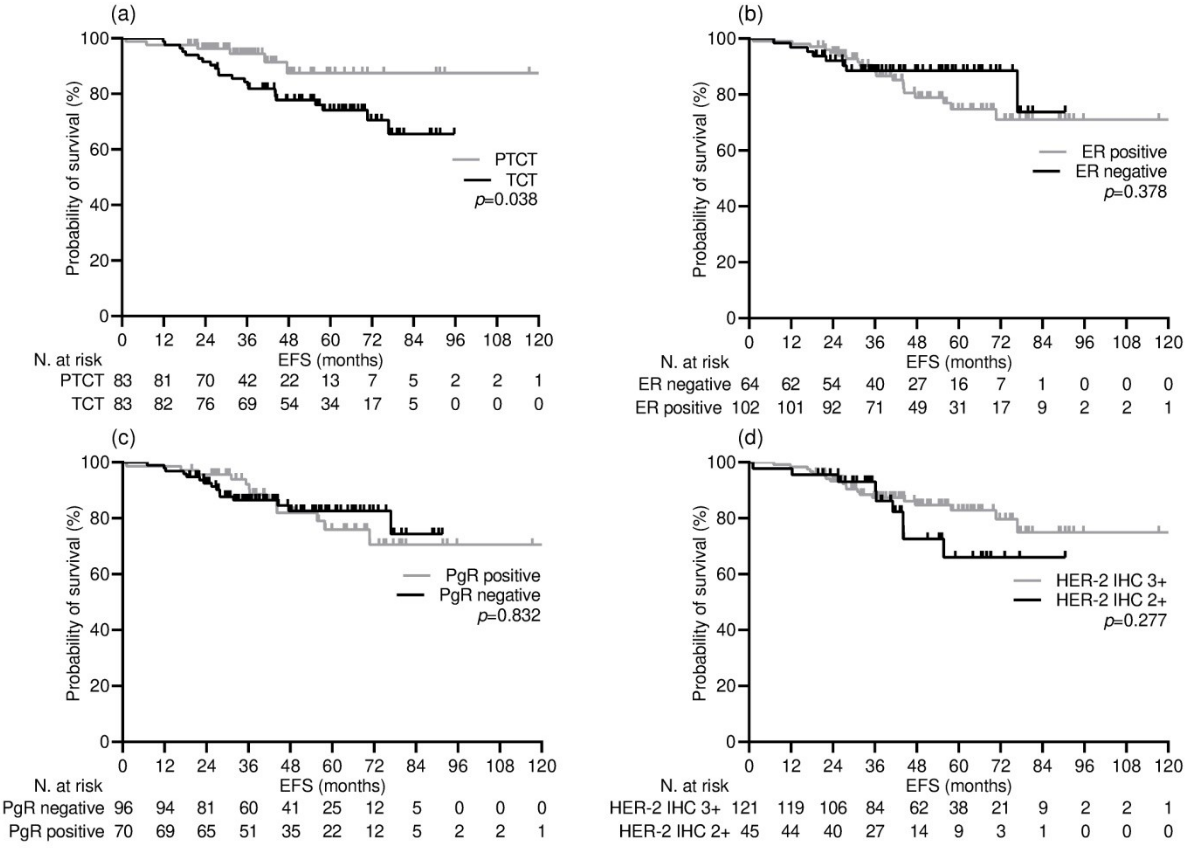

PSM, propensity score matching; (a) type of neoadjuvant treatment: pertuzumab-trastuzumab-chemotherapy (PTCT) vs trastuzumab-chemotherapy (TCT); (b) estrogen receptor (ER) status: positive vs negative; (c) progesterone receptor (PgR) status: positive vs negative; (d) human epidermal growth factor receptor 2 (HER-2) scoring at immunohistochemistry (IHC): 3+ vs 2+

Supplementary Figure 3. Event free survival by lymph node status after PSM

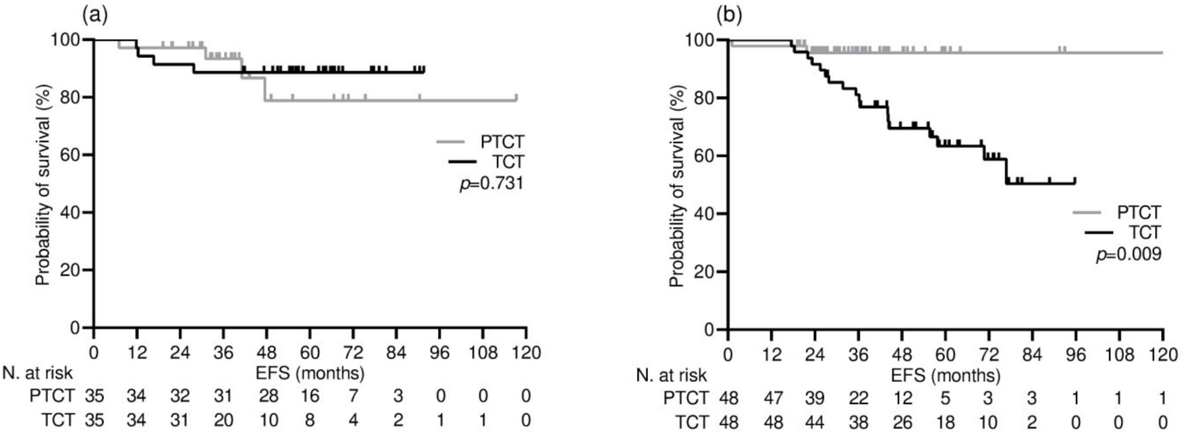

PSM, propensity score matching; (a) clinically lymph node negative disease (cN0); (b) clinically lymph node positive disease (cN+); PTCT, pertuzumab-trastuzumab-chemotherapy; TCT, trastuzumab-chemotherapy
